# Catatonia induced by nitrous oxide anesthesia in a healthy adolescent: A case report

**DOI:** 10.1002/pcn5.70189

**Published:** 2025-08-13

**Authors:** Kenji S. Kobayashi, Takuto Ishida, Hitomi Tsunashima, Takero Terayama, Eiken Yoshida, Masafumi Mizuno

**Affiliations:** ^1^ Department of Psychiatry Tokyo Metropolitan Matsuzawa Hospital Tokyo Japan; ^2^ Department of Psychiatry and Behavioral Science Juntendo University Graduate School of Medicine Tokyo Japan; ^3^ Department of Internal Medicine Tokyo Metropolitan Matsuzawa Hospital Tokyo Japan; ^4^ Department of Emergency Medicine Self‐Defense Forces Central Hospital Tokyo Japan; ^5^ Department of Emergency Medicine National Hospital Organization Tokyo Medical Center Tokyo Japan; ^6^ Present address: Asaka Hospital Fukushima Japan

**Keywords:** benzodiazepine, catatonia, nitrous oxide, refractory catatonia, vitamin B12

## Abstract

**Background:**

Catatonia can arise from various conditions, not only psychiatric but also non‐psychiatric etiologies. This diversity of underlying causes makes it challenging to identify the underlying etiology, which is crucial for appropriate management. Nitrous oxide (N_2_O) is widely used for recreational purposes, and its adverse effects have become a growing concern. Chronic exposure can lead to vitamin B12 deficiency, which manifests both neurological and psychiatric symptoms, including catatonia. Vitamin B12 supplementation is an effective treatment, but the diverse clinical manifestations of N_2_O toxicity, coupled with the diverse etiologies of catatonia, can delay diagnosis and intervention.

**Case Presentation:**

The patient, a healthy man in his 20s, had been receiving N_2_O anesthesia regularly for pain relief while undergoing cosmetic hair removal. After his seventh inhalation, he developed a catatonia and was admitted to a previous hospital. After a comprehensive examination failed to identify the etiology, he was transferred to our hospital for psychiatric assessment. Intravenous benzodiazepine failed to improve his catatonia. Re‐evaluation of the underlying etiologies of benzodiazepine‐refractory catatonia raised the possibility of a vitamin B12 deficiency resulting from chronic N_2_O exposure. Vitamin B12 supplementation promptly improved his catatonia, and the patient was discharged without any recurrence of his symptoms.

**Conclusion:**

Catatonia developed in the present patient after multiple exposures to N_2_O. Active vitamin B12 may be administered if N_2_O is suspected of causing psychotic symptoms. Moreover, reassessing the differential diagnosis is worthwhile when managing refractory catatonia.

## BACKGROUND

Catatonia is a neuropsychiatric syndrome characterized by abnormal behavior, movements, and withdrawal.^1^ This condition was formerly classified as a subtype of schizophrenia but is now known to be caused by physical disorders, such as autoimmune encephalitis and viral meningitis, as well as by drugs and medications.^2^, ^3^ Benzodiazepines are the first‐line treatment for catatonia; however, there is currently no consensus on the management of benzodiazepine‐refractory catatonia.^3^ The decision either to proceed with electroconvulsive therapy (ECT) or to prioritize a more thorough reassessment of the underlying causes is left to the clinician's discretion.

Nitrous oxide (N_2_O), a traditional inhalation anesthetic, is commonly referred to as laughing gas due to its euphoric side effects. N_2_O is increasingly being used for recreational purposes worldwide, raising concerns about its potential side effects.^4^ On the other hand, the medical use of N_2_O has generally declined; the gas is now primarily limited to dentistry and cosmetic procedures, including hair removal procedure. Chronic exposure to N_2_O results in various clinical presentations, most notably neurological complications and psychiatric symptoms.^5^, ^6^ Reported cases include presentations mimicking acute meningitis,^7^ as well as episodes of acute psychosis.^8^, ^9^ The diversity of the presentations may be attributed to the irreversible inactivation of vitamin B12 by N_2_O.^10^, ^11^ Vitamin B12 deficiency is linked to various psychiatric symptoms,^12^ including acute psychosis and catatonia.^13^, ^14^ Vitamin B12 supplementation is thus crucial for treating chronic N_2_O exposure^5^, ^6^; however, the wide range of clinical backgrounds and manifestations can lead to misdiagnoses and delays in appropriate treatment.

Herein, we report a case of a healthy adult male individual in whom benzodiazepine‐refractory catatonia developed after N_2_O anesthesia, and fully recovered after vitamin B12 administration.

## CASE PRESENTATION

A previously healthy male patient in his 20s presenting with catatonia was referred to our hospital on suspicion of psychiatric disorders. He had attended a private mental health clinic for a brief period approximately 3 years prior to current presentation for treatment of work‐related anxiety, but had no other significant medical or psychiatric history. He had no family history of any psychiatric disorder. He was not receiving any medication regularly, and no history of illicit drug use except for a single instance of marijuana use during a trip abroad several years prior. He had no particular dietary preferences.

The patient had been visiting a cosmetic clinic for hair removal about once every three months for a year and received inhalational anesthesia with 40% N_2_O for about 30 min to relieve the pain caused by the procedure. Three days before his admission to our hospital, he underwent his seventh procedure under N_2_O anesthesia as usual. During the inhalation, he complained of discomfort, fixed his gaze in a blank stare, and began loudly repeating the name of the color that he was apparently seeing. He was transferred to a nearby emergency hospital, and his symptoms improved with pure oxygen inhalation. However, the symptoms recurred after the patient returned home, leading to admission to the emergency department of the same hospital for further evaluation 2 days before his current admission.

The results of comprehensive investigations, including blood and cerebrospinal fluid analyses, whole‐body computed tomography (CT), and contrast‐enhanced brain magnetic resonance imaging (MRI), were unremarkable. The serum levels of vitamin B1 and B12 after a vitamin B complex injection (thiamine hydrochloride 100 mg, pyridoxine hydrochloride 100 mg, and cyano‐cobalamin 1 mg) were in the normal range (vitamin B1: 52.5 ng/mL; vitamin B12: 3,040 pg/mL). A screening test for the SARS‐CoV‐2 antigen returned positive, but his respiratory symptoms were unremarkable. A SARS‐CoV‐2 PCR test using cerebrospinal fluid returned negative. During hospitalization, he sometimes exhibited multiple abnormal movements, kept shouting the same word or swung his legs violently from his bed; at other times he became unresponsive and stared into space. Diazepam and haloperidol were administered for sedation; however, these symptoms did not improve.

Given the persistence of the symptoms and the absence of abnormal findings on comprehensive investigations, he was referred to our hospital for a suspected psychiatric disorder. Upon arrival, his vital signs were unremarkable except for tachycardia. While being transported on a stretcher, he exhibited vigorous thrashing movements and repeatedly uttered certain phrases; when asked his name, he responded by incessantly repeating it, as he did with other words that he had heard. When his arm was passively raised, he maintained the position. Based on these symptoms, we suspected him of catatonia and administered a slow intravenous injection of midazolam for sedation and diagnostic treatment. Thereafter, he fell asleep without any improvement in communication. Blood tests at admission demonstrated hemoglobin 14.1 g/dL and mean corpuscular volume 91.7 fl, both of which were within the normal range. A urine toxicology test (SIGNIFY™ ER) returned positive only for benzodiazepine, which had been administered at the previous hospital.

After admission, we initially continued administering diazepam 10 mg/day and haloperidol 5 mg/day intravenously, both of which had also been used at the previous hospital. On hospital Day 2, the patient continued to exhibit disturbing behavior and loudly to repeat phrases such as “Fifty is good, twenty‐five is bad.” He clung to the railing of the bed and did not respond to any inquiries. His catatonia persisted despite 3 days of benzodiazepine administration; therefore, other treatment options, including ECT, were considered. However, given the absence of a significant psychiatric history and his exposure to N_2_O immediately prior to symptom onset, N_2_O was reconsidered as the possible cause of the catatonia, despite no history of N_2_O abuse. Consequently, 500 μg of mecobalamin was intravenously administered on the evening of hospital Day 2 for N_2_O intoxication. On the morning of hospital Day 3, his speech had normalized, and he said, “I couldn't distinguish between dream and reality.” On hospital Day 4, his family visited the patient and were reassured that he returned to his usual state. Electroencephalography conducted on hospital Day 9 demonstrated no epileptiform spikes or slow waves. A comprehensive screening test of anti‐neuronal antibodies in the cerebrospinal fluid, including anti‐NMDA receptor antibody, yielded negative results. Mecobalamin 1500 µg/day was continued, and the diazepam and haloperidol dosage was gradually decreased. The patient did not exhibit any further symptoms and was discharged from the hospital on Day 13.

The patient received follow‐up at our outpatient clinic for several months without exhibiting any catatonic symptoms following the discontinuation of psychotropic therapy.

## DISCUSSION

The present patient exhibited catatonia after multiple exposures to N_2_O without any episode of abuse. Repeated treatment with benzodiazepines failed to improve his catatonia, whereas the administration of vitamin B12 led to a dramatic recovery. The clinical course of this patient illustrates two important points. First, N_2_O causes psychiatric adverse effects, including catatonia, regardless of a history of N_2_O abuse. Second, potential causes of catatonia should be thoroughly assessed in patients whose symptoms are refractory to benzodiazepine treatment.

The recreational use of N_2_O has recently been increasing worldwide.^4^ Its unregulated and inappropriate use outside the medical settings has revealed a variety of adverse effects that are not typically observed in a controlled medical environment. While the neurotoxicity of N_2_O has long been known,^10^ it is important to note that psychiatric symptoms, such as hallucinations and delusions, can also occur as side effects.^15^, ^16^ Notably, psychiatric symptoms can occur even with appropriate N_2_O administration by medical professionals, as in this case. The psychiatric symptoms are primarily explained by the interaction of N_2_O with vitamin B12 metabolism.^5^, ^6^, ^16^ N_2_O irreversibly oxidizes the cobalt ion in vitamin B12, leading to inactivation and disruption of critical enzymatic reactions, including methionine synthase activity.^10^ Thus, vitamin B12 supplementation is the first‐line treatment for N_2_O intoxication.^5^, ^6^ The association between vitamin B12 deficiency and catatonia has been observed in several previous case reports.^13^, ^17^, ^18^, ^19^, ^20^, ^21^, ^22^ In many of these cases, the patients were treated with a combination of vitamin B12 and psychotropic medications,^18^, ^19^, ^20^, ^21^, ^22^ but in one case, the patients recovered completely with vitamin B12 supplementation alone.^17^ To the best of our knowledge, the present case is the second documented instance of N_2_O‐induced catatonia.^22^ The first case developed catatonia following long‐term abuse whereas the present patient experienced the sudden onset of catatonia after occasional N_2_O use under medical supervision. Furthermore, our patient achieved complete remission of benzodiazepine‐refractory catatonia following vitamin B12 administration. This case thus presented an uncommon side effect of N_2_O inhalation, emphasizing the need for greater awareness of this potential complication whatever the patient's exposure history.

In chronic N_2_O exposure, the serum vitamin B12 level often appears normal despite the presence of symptoms.^6^ This discrepancy arises because routine blood test measures cyano‐cobalamin, the inactive form of vitamin B12, which does not accurately reflect active vitamin B12 deficiency. Additional tests for urinary methylmalonic acid and serum homocysteine are more useful for assessing active vitamin B12 deficiency.^23^ In the present case, the patient's serum vitamin B12 level was high at 3040 pg/mL because it was measured after administering cyano‐cobalamin‐containing intravenous injections at the previous hospital. The active vitamin B12 deficiency in the patient was not quantified because additional measurements of urinary methylmalonic acid and serum homocysteine were unable to be performed. Although vitamin B12 supplementation is recommended for the treatment of N₂O intoxication,^6^ it remains unclear based on previous reports and clinical guidelines whether the active or inactive form should be used. In this case, we selected methyl‐cobalamin supplementation, an active form of vitamin B12, based on two considerations: (1) the biologically active forms of vitamin B12 in the human body are methyl‐cobalamin and adenosyl‐cobalamin, and N₂O toxicity specifically impairs methyl‐cobalamin‐dependent pathways^24^; and (2) the patient's symptoms had not improved despite the administration of cyano‐cobalamin. Following the administration of methyl‐cobalamin, the patient exhibited a dramatic improvement in catatonic symptoms in a short period. This clinical response supports the hypothesis that a functional deficiency in active vitamin B12 was still present at the time of admission. Based on the above, it can be inferred that the catatonic state of the patient was precipitated by a dysfunction of active vitamin B12 caused by N_2_O inhalation.

Previous reviews of N_2_O abuse leading to psychiatric symptoms^15^, ^16^ have reported that the frequency of inhalation ranged from once a week to daily, over several months to more than a year. However, one report described a case of depression following just four days of exposure.^25^ In the present case, 40% N_2_O was inhaled on each occasion for approximately 30 min. While this amount may not appear substantial, the total exposure seven times inhalation over the course of about a year might be sufficiently prolonged to contribute to psychiatric adverse effects. Given that the cumulative amount of inhaled N_2_O cannot be quantified, it is crucial to consider N_2_O exposure as a potential cause of psychiatric symptoms in any patient with a history of its use, regardless of the frequency or amount of use.

The specific risk factors contributing to the development of psychiatric symptoms following N_2_O inhalation remain poorly understood. This may be because, unlike the present case, many of the previously reported cases already had a substantial background of nitrous oxide abuse. Paulus et al.^16^ reviewed the cases in which individuals developed psychiatric symptoms following recreational N_2_O use, and reported that 11 out of 31 cases had a history of psychiatric disorders. This finding suggests that a prior history of psychiatric disorders may constitute an important risk factor for the development of psychiatric symptoms in the context of N_2_O exposure.

Catatonia can arise from psychiatric or non‐psychiatric conditions, which are broadly categorized into central nervous system‐related disorders, drug or medication use or withdrawal, and other conditions.^26^, ^27^ Although benzodiazepines are recommended as the first‐line treatment,^3^, ^28^ their efficacy may be limited.^29^ ECT is generally considered as the second‐line option^3^, ^28^; however, the treatment algorithm presumes that physical etiologies have been ruled out. In practice, non‐psychiatric causes of catatonia often involve rare conditions,^26^ hence, these underlying diseases can be overlooked. In cases of refractory catatonia, the decision to initiate ECT or to reassess the patient for rare physical causes is left to the clinician's discretion. In the present case, the initial investigations revealed no clear physical etiology, and benzodiazepines were ineffective. Given the presence of features consistent with medical catatonia as described by Oldham^26^ and a history of N_2_O exposure, we prioritized physical reassessment over ECT. This case may be an example of the need for a thorough diagnostic work‐up prior to ECT in cases of refractory catatonia.

## CONCLUSION

In conclusion, active vitamin B12 administration should be considered as a treatment for psychiatric symptoms in a patient with a history of N_2_O exposure even if the exposure occurred under medical supervision. When treating patients with refractory catatonia, it may be worth reassessing for potential physical causes before proceeding to ECT.

## AUTHOR CONTRIBUTIONS

Kenji S. Kobayashi, Takuto Ishida, Hitomi Tsunashima, Takero Terayama, and Eiken Yoshida treated the patient. Kenji S. Kobayashi wrote the first draft of this article. The manuscript was mainly reviewed by Takuto Ishida. All the authors have read and approved the final version of this manuscript.

## CONFLICT OF INTEREST STATEMENT

The authors delcare no conflicts of interest.

## ETHICS APPROVAL STATEMENT

N/A.

## PATIENT CONSENT STATEMENT

Written informed consent was obtained from the patient for the publication of this case report.

## CLINICAL TRIAL REGISTRATION

N/A.

## Data Availability

The data that support the findings of this study are available from the corresponding author upon reasonable request.
